# Menstrual Hygiene, Management, and Waste Disposal: Practices and Challenges Faced by Girls/Women of Developing Countries

**DOI:** 10.1155/2018/1730964

**Published:** 2018-02-20

**Authors:** Rajanbir Kaur, Kanwaljit Kaur, Rajinder Kaur

**Affiliations:** ^1^Department of Botanical and Environmental Sciences, Guru Nanak Dev University, Amritsar, Punjab 143005, India; ^2^Govt. Polytechnic College for Girls, Amritsar, India

## Abstract

Menstruation and menstrual practices still face many social, cultural, and religious restrictions which are a big barrier in the path of menstrual hygiene management. In many parts of the country especially in rural areas girls are not prepared and aware about menstruation so they face many difficulties and challenges at home, schools, and work places. While reviewing literature, we found that little, inaccurate, or incomplete knowledge about menstruation is a great hindrance in the path of personal and menstrual hygiene management. Girls and women have very less or no knowledge about reproductive tract infections caused due to ignorance of personal hygiene during menstruation time. In rural areas, women do not have access to sanitary products or they know very little about the types and method of using them or are unable to afford such products due to high cost. So, they mostly rely on reusable cloth pads which they wash and use again. Needs and requirements of the adolescent girls and women are ignored despite the fact that there are major developments in the area of water and sanitation. Women manage menstruation differently when they are at home or outside; at homes, they dispose of menstrual products in domestic wastes and in public toilets and they flush them in the toilets without knowing the consequences of choking. So, there should be a need to educate and make them aware about the environmental pollution and health hazards associated with them. Implementation of modern techniques like incineration can help to reduce the waste. Also, awareness should be created to emphasize the use of reusable sanitary products or the natural sanitary products made from materials like banana fibre, bamboo fibre, sea sponges, water hyacinth, and so on.

## 1. Introduction

According to World Health Organization, a person aged 10–19 years is considered as an adolescent [1]. The transition period between the childhood and adulthood is called adolescence which is marked with the growth and development of the child. During this period, physical, psychological, and biological development of the child occurs [2]. It is recognized as a special period in a girl's life cycle which requires special attention. Menarche is an important biological milestone in a woman's life as it marks the onset of the reproductive phase of her life. The average age at menarche is mostly consistent across the populations, that is, between 12 and 13 years of age [3, 4]. Unfortunately, due to lack of knowledge on menstruation preparedness and management or due to shyness and embarrassment the situation becomes worse for girls [5]. Menstruation is a natural process but it is still a taboo in Indian society as it is considered unclean and dirty [6].

Menstruation wastes are the wastes that are generated by a female in her reproductive years. These wastes are produced during menstruation commonly known as menses, periods, or monthly bleeding cycle [7]. The menstrual cycle has three phases, that is, follicular phase (proliferative), ovulation phase, and luteal phase (secretory). Menstruation is regulated by hormones; in this process, endometrium, lining of uterus, gradually thickens and sheds off and causes bleeding that normally last for 3–5 days and occasionally up to 7 days. Menstruation sheds two-thirds of the endometrial lining. In addition to blood, menstrual fluid contains mucus and vaginal secretions [8]. The menstrual flow varies from female to female and may be more or less at the beginning of menses or may change throughout the cycle. The color of the menstrual fluid varies between red, bright red, and dark brown to black. Menstrual fluid may or may not have unpleasant odour especially when it comes in contact with air. Menstrual flow or duration also changes before menopause or during gynaecological cancers. Under conditions of hormonal imbalance, fibroids, polyps, and endometriosis menstrual flow increase and excessive loss of blood through menstruation can lead to anaemia.

Women have developed their own personal strategies to handle this period of time. Globally, these strategies vary greatly due to the personal preferences, availability of resources, economic status, cultural traditions and beliefs, education status, and knowledge about menstruation. Practices related to menstruation hygiene are of major concern as it has a health impact; if neglected, it leads to toxic shock syndrome, reproductive tract infections (RTI), and other vaginal diseases [9–11]. Poor genital hygiene negatively affects adolescents' health. Most girls are unaware and unprepared for menarche as they are not informed or ill-informed about menstruation [12]. The main objective of this review was to summarize the concern and possible methods of menstrual waste management in low-income countries. The review article was aimed at understanding the menstrual practices, product design, demands, and disposal strategies. It includes both a summary of the existing menstrual hygiene needs and management and also an analysis of the current knowledge in the fields of public health, water and sanitation, and solid waste management.

## 2. Cultural Beliefs and Restrictions during Menstruation

Menstrual hygiene practices were affected by cultural norms, parental influence, personal preferences, economic status, and socioeconomic pressures. Menstrual beliefs refer to misconceptions and attitudes towards menstruation within a given culture or religion. Menstrual beliefs, knowledge, and practices were all interrelated to the menstrual hygiene management [13, 14]. By reviewing literature and articles published in journals and reports available on the Internet we found many cultural and religious beliefs followed by people regarding menstruation. These norms were the barriers in the path of good menstrual hygiene practices. Many women experiencing restrictions on cooking, work activities, sexual intercourse, bathing, worshipping, and eating certain foods [15]. These restrictions were due to the overall perception of the people regarding menstruation as they consider it dirty and polluting [16].

In some parts of the country there were restrictions on bathing and a taboo against burial of bloodied menstrual cloth. Cloths should first be washed and then buried or reused. Washing and drying thought to be done secretly or in a hidden corner so that it cannot be seen by others [17]. It was also believed that menstrual fluids may be misused for black magic, so women should wash the wrapper/cloth wore during menses only at night when others were asleep [18]. Menstrual flow was seen as dirty, polluting, and shameful, so women hide menstrual cloths for fear of being cursed. In similar findings, it was believed that menstrual waste was linked to witchcraft and danger, so it must be buried unless witches go after human blood and find the menstrual wrapper/cloth and destroy the women by causing infertility [13]. From all these beliefs, it was clear that education plays a key role in menstruation hygiene management. By educating both men and women regarding menstruation, we can overcome these false beliefs and taboos. Due to cultural expectations and restrictions many girls were not adequately informed about the realities of menstruation. As a result, they feel subnormal, diseased, or traumatized [19]. Unprepared girls were frightened, confused, and feel embarrassed by menarche likely to develop negative attitudes towards menstruation [20]. Even touching of menstruating women was considered toxic, they were prohibited from cooking and from taking certain foods like pickle. These prohibitions are more in the rural areas than in the urban areas. They were also not allowed to participate in religious activities or to contact religious articles [21]. Menstruating girls are also not allowed to bath and wash hair, as it is believed to impede blood flow.

## 3. Types of Absorbents Used during Menstruation

**Figure 1:**
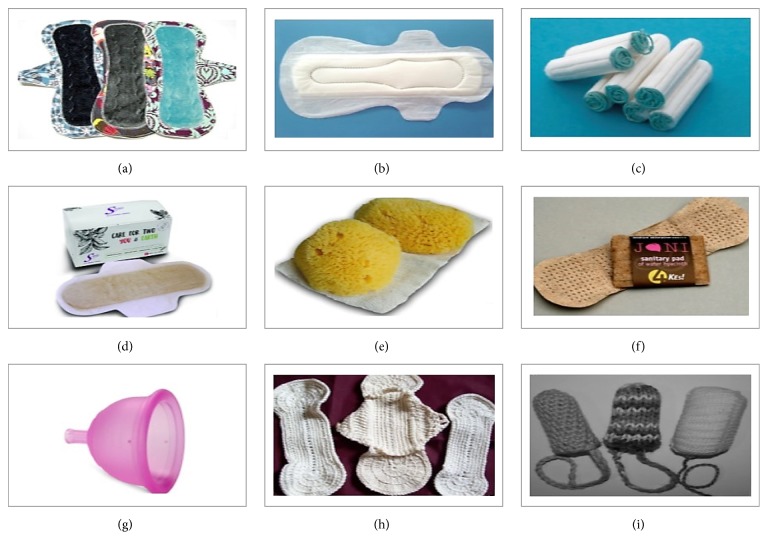
Types of sanitary products used by women during menstruation are (a) reusable cloth pads (https://www.etsy.com/market/cloth_menstrual_pads); (b) commercial sanitary pads (http://topyaps.com/things-girl-must-know-about-sanitary-pads); (c) tampons (http://www.womensvoices.org/tag/tampons/); (d) pads made from banana fibre (https://saathipads.com/); (e) sea sponges used as sanitary material (https://www.pinterest.com/pin/194640015120225878/); (f) pads made up of water hyacinth (https://www.ecouterre.com/jani-a-biodegradable-sanitary-napkin-made-from-water-hyacinth/); (g) menstrual cup (http://rubycup.com/blog/how-to-clean-the-suction-holes-of-your-menstrual-cup/); (h) pads made from wool (https://www.pinterest.com/pin/198088083583361670/); (i) reusable tampons (http://naturalparentsnetwork.com/reusable-menstrual-products/).

The preference of sanitary protection material is based on personal choice, cultural acceptability, economic status, and availability in local market. Along with basic sanitation facilities, one should be also provided with soap and menstrual absorbents to manage menstruation hygiene. The choice of absorbents varies among rural and urban women and girls. In rural areas, the most preferred absorbents are reusable cloth pads and in urban areas women prefer to use commercial sanitary pads. Chlorine-bleached Kraft or sulphate pulp is used by manufacturers to produce fluff pulp as absorbent used to make disposable sanitary products. Nowadays, many deodorised and non-deodorised sanitary products are available in the market made of synthetic fibre rayon. These deodorised products contain chemicals like organochlorines which have antibacterial activity. Due to their chemical composition, these products when buried in the soil they kill the soils microflora and delay the process of decomposition [22]. Different menstrual products used by women/girls are discussed below (Figure 1).

### 3.1. Reusable and Washable Cloth Pads

They may be sustainable sanitary option but must be hygienically washed and dried in the sunlight. The sun's heat is a natural sterilizer and drying the cloths/cloth pads under it sterilizes them for future use. These cloth pads are reusable so they are cost-effective, easily available, and ecofriendly. They also need to be stored in a clean dry place for reuse to avoid contamination.

### 3.2. Commercial Sanitary Pads

They are easily available at many stores, chemist shops, or online. They are expensive, compared to cloth pads, nonreusable, and not very environment-friendly. The cotton used in their making is not 100% natural and may contain pesticides.

### 3.3. Tampons

They are the type of absorbent that provides internal protection. They are kind of plug of soft material (cotton) which is inserted into the vagina to absorb the menstrual flow before it leaves the body. They are expensive, not easily degradable in nature and, hence, not very environmental friendly. Nowadays, sea sponge tampons are available in the market which are a natural alternative to synthetic tampons.

### 3.4. Reusable Tampons

These are washable tampons made up of natural materials like bamboo, wool, cotton, or hemp. They are also knitted or crocheted using the natural absorbent material like cotton or wool. They are inserted into the vagina to absorb menstrual flow same as the disposable tampons.

### 3.5. Menstrual Cups

They may be a new technology for poor women and girls and an alternative to sanitary pads and tampons. They are like cups made of medical grade silicone rubber which makes the cup easy to fold and get inserted into the vagina to collect menstrual blood. They can be worn up to 6–12 hours depending upon the amount of menstrual flow, so it needs to be removed and emptied less frequently. They are reusable and environment-friendly. It offers sustainable, practical, and cost-effective alternative where sanitation conditions are not good.

### 3.6. Bamboo Fibre Pads

Instead of wood pulp, bamboo pulp is used as an absorbing material in these sanitary pads. It has more absorbing capacity and is safer to use. They are affordable, easily decomposed, and environment-friendly pads which also possess antibacterial properties. This provides infection and irritation-free menstruation. Also, bamboo charcoal pads are available in the market with advantage that blood stains are not clearly visible and are also reusable in nature.

### 3.7. Banana Fibre Pads

Nowadays, low-cost sanitary pads for rural women made from waste banana tree fibre were sold under trade name “Saathi” in India. They are environment-friendly and decompose within six months after use. Besides these products, women in the remote rural areas also use natural materials like cow dung, leaves, and mud (https://sswm.info/category/background/background/background/health-and-hygiene-issues/menstrual-hygiene-management).

### 3.8. Water Hyacinth Pads

Menstrual pads manufactured using water hyacinth is sold under trade name “Jani.” They are cost-effective, easily biodegradable, and ecofriendly in nature.

## 4. Menstrual Waste Disposal Techniques Used by Women

Appropriate disposal of used menstrual material is still lacking in many countries of the world. Most of the countries have developed techniques to manage their fecal and urinary wastes but, because of lack of menstrual management practices in the world, most of the women dispose of their sanitary pads or other menstrual articles into domestic solid wastes or garbage bins that ultimately become a part of solid wastes. Toilet facilities in India lack bins for the disposal of sanitary pads and hand washing facilities for menstruating women to handle menstrual hygiene. In urban areas, where modern disposable menstrual products are used they dispose of them by flushing in toilets and throwing in dustbins or through solid waste management [23], but, in rural areas, there are many options for disposing menstrual waste such as by burying, burning, and throwing in garbage or in pit latrines. In rural areas, mostly women use reusable and non-commercial sanitary materials like reusable pads or cloths. Thus, they generate lesser amount of menstrual waste as compared to women in urban areas who rely on commercial disposable pads. The menstrual material was disposed of according to the type of product used, cultural beliefs, and location of disposal. In slum areas, women dispose their menstrual waste into pit latrines as burning and burial were difficult due to limited privacy space [24]. The reason behind that is it was seen by men or used in witchcraft.

In schools, due to lack of sanitary facilities, girls throw their pads in toilets. In some cases, girls threw away their used menstrual clothes without washing them. Also many were reported being absent from school due to lack of disposal system, broken lock/doors of toilets, lack of water tap, bucket, and poor water supply [25, 26]. In some schools, incinerators or “feminine hygiene bins” are used for disposing menstrual waste material but due to shyness or fear of being seen by others they refrained from using it [27]. The behavior of women regarding disposal is different when being at home and away from home. At home, they dispose the waste by wrapping and throwing in the dustbin along with other domestic waste. As mentioned above, the disposing habits change according to the place. In public places, prior to having knowledge about the consequences of flushing the pads, they flush them in the toilets or wrap and throw them in the dustbins. Where dustbins are not placed they leave the soiled pads wrapped or unwrapped in the toilet corners. This makes the toilets dirty, breeding place for flies and mosquitoes, and also unhygienic for other toilet users and cleaners. In many cities, the persons who manage the public toilets always complain of blockage of sewage system because of flushing of sanitary pads or rags in the toilet.

## 5. Consequences of Menstrual Waste Disposal

As sanitation systems were designed with urine and feces in mind, they are unable to cope with the menstrual absorption materials. These absorption materials clog the sewer pipelines as they are unable to pass through and cause the system backflow [28]. Materials like tampons, cotton wool, toilet paper, and other organic materials used for menstrual management might be decomposed in pit latrines/landfills except the plastic inlay of the commercial sanitary pads. Sanitary napkins might decompose over a period of about one year except its plastic lining in on-site sanitation [29].

In rural areas, pit latrines once full they were covered with soil and new pit was dug but due to space limitations this was not practiced in urban areas [30]. It was reported that some women and girls wrap their used menstrual cloths and packs in polythene bags before disposing in pit latrines which prevents them from decomposition. Nowadays, mostly women/girls prefer commercial sanitary pads and tampons which are made up of superabsorptive materials like polyacrylate. These pads and tampons when flushed in the toilets they get saturated with liquid and swell up, thus resulting in sewage backflow, a serious health hazard. The adhesive wings and the perforated plastic layers in the commercial sanitary napkins are not easily biodegradable. The sewage blockages were mostly due to accumulation of excessive quantity of solid waste or sand which results in hardening of the sludge in the pits. Blockage of sewage system is a global problem and major contributing factor is flushing of menstrual products in toilets. Deodorised sanitary products used by women/girls contain chemicals used in bleaching such as organochlorines which when buried in the soil disturb the soil microflora and decomposition takes time [22].

People living alongside river banks throw menstrual waste into water bodies which contaminate them. These materials soaked with blood were breeding places for germs and pathogenic microbes [31]. Sanitary products soaked with blood of an infected women/girl may contain hepatitis and HIV viruses which retain their infectivity in soil and live up to six months in soil. The clogged drainage with napkins has to be unblocked and cleaned manually by conservancy workers with their bare hands without proper protection and tools. This exposes the workers to harmful chemicals and pathogens. Incineration is a better technique to dispose of menstrual waste but burning of pads releases harmful gasses that effects health and environment. Burning of inorganic material at low temperature releases dioxins which are toxic and carcinogenic in nature.

## 6. Role of Men/Boys towards Menstrual Hygiene Management

One of the main reasons why menstruation is a taboo and menstruation hygiene is neglected is gender inequality. Unequal rights given to men and women result in women's voices being ignored within households and communities and in development programmes. Due to cultural norms and stigmas, menstruating women are not allowed to use water and sanitation facilities and in some cases even excluded from home as menstruation is considered impure [32, 33]. Therefore, comprehensive programmes that engage both men and women should be organized related to menstrual hygiene.

Men can support and influence women and girls in managing menstruation in households, schools, work, and community through many roles as husbands, fathers, brothers, students, teachers, colleagues, leaders, and policymakers. By reviewing literature, it was found that at household level men do not support women regarding menstruation hygiene and never have they discussed menstrual issues with their wives and daughters. As they are decision-makers at household level, in many cases they do not give money to buy menstrual products such as commercial sanitary pads, tampons, and menstrual cups as they consider it money wastage. So women have to rely on cheap reusable cloth pads which they have to wash, dry, and use again. In other cases, due to low family income, men hesitate to give money for such costly products. So, in both cases, women have to compromise with their menstrual needs and personal hygiene [34].

Decisions related to constructing toilets in houses are also taken by male members. So it is a big barrier in menstrual hygiene as women find it difficult in cleaning and changing menstrual materials in privacy [35]. Women and girls who have toilets at home feel shy and embarrassed as the drain that leads out is not covered and there is a chance of seeing blood flowing in drain by others [36, 37]. In some reports, it was found that parents did not allow boys to discuss such topic as they were not important for their future, so the boys received information about menstruation from friends and Internet which is inaccurate and incomplete. In some places, like in Nepal, menstruating women have to live separately in a “chhaupadi” during menstruation outside the house. This cultural norm is supported by both men and women making it difficult to survive for a menstruating women/girl during winters [38]. They also have to face many challenges living in chhaupadi's such as getting bitten by snakes, fires, and rapes.

Most men do not know about the menstruation and physiological changes in women during menstruation and menstrual cycle, so it is difficult to change their perception regarding menstruation and menstrual hygiene. Due to unwillingness, myths, prejudices, and misconceptions, it is difficult to talk about menstruation with men and boys. But by engaging them into group discussions and regular community meetings, we can change their perception and make them aware about their role regarding menstrual hygiene management. In India, a man named “Arunachalam Muruganantham” known as “India's Menstrual Man” develops an inexpensive and environment-friendly machine which produces semibiodegradable sanitary pads. Men can help women and girls by constructing toilets, incinerators, and latrines with chutes at homes and schools and at community level. At household level, they help by providing toilet facilities with privacy, water, and soap and by giving them money for menstrual products. As the decision-making power is in men's hand, making household budgeting for sanitary materials supports and empowers women by allowing them to move freely with lower risk of stains. Men who are in politics support menstrual hygiene management by making girls/women friendly policies, by providing sanitary materials free of cost or at affordable prices, by providing water and sanitation in their areas, and by conducting seminars and workshops in rural areas.

## 7. Role of Teachers in Creating Awareness regarding Menstruation and Menstrual Waste Management

In schools, teachers can make the school environment girl/women friendly to manage menstruation with dignity. Sex education in schools helps adolescents to discover their sexual identity, to protect themselves from sexual abuse, unwanted pregnancies, and sexually transmitted diseases, and to know physiological changes occurring in the body and how to take care of personal hygiene [39]. In most of the cases, teacher's attitude is not good and supportive towards menstruating girls in schools. Different views of parents, teachers, and society affect sex education being taught in schools and colleges. Cultural, religious, and social barriers also create hindrance in the path of sex education [40].

Our education sector plays an important role in child's growth and development by allowing them to respond to changes and challenges they are facing in day-to-day life [41]. But many times it avoids issues related to the menstruation and menstrual hygiene management by considering it one's personal matter and should be discussed within the house. Menstruation is a silent issue in girl's life which is further affected by teacher's attitude, school environment, and infrastructure. Because of this. many girls remain absent from schools during this time. Sex education is often neglected from the school curriculum which negatively impacts the student's life. They get information about puberty, sexual intercourse, menstruation, and other physiological changes in one's body from books, friends, and Internet which may be incomplete or inaccurate. Due to lack of knowledge and social interaction, teasing and taunting with hurtful nicknames are common in schools. This makes it difficult for a girl student to survive in this environment, so they remain absent from school.

In many schools, both male and female teachers are not ready to discuss menstruation and menstrual hygiene management with students. The female teachers are also not available in many schools. Teachers often skip such topics in books as they do not want any open discussion in the class or to escape from the questions asked by students. Teachers also feel shy and embarrassed to discuss such topics in class due to language barrier [42]. In most schools, English is not a compulsory subject so teachers have to discuss them in local language and using vernacular words in front of students is an embarrassing thing. Due to unsupportive environment in the schools, it was also found that some girls hesitate to stand to answer teacher's questions in fear of leakage or smell and also hesitate to write on blackboard in fear of any menstrual accident and blood stains on clothes seeing by others. In some reported cases, parents do not allow girls to go to schools upon reaching puberty in fear of sexual harassment by boys and male teachers in schools [43].

To overcome these issues, male teachers and employees in the schools and institutes should be well educated and confident regarding menstruation and menstrual hygiene management so that they support girls/women by providing safe environment and privacy. A committee of teachers including both male and females should be made in the schools to collect funds for providing sanitary napkins, soaps, water, and toilet facilities in schools so that girls manage their menstruation with ease and safety. Committee should also provide dustbins for menstrual waste disposal. Separate toilets for girls and boys with proper doors and locks should be built in the schools. Teachers should educate girls about menstrual health management and its link to their health. They should also make girl students aware of how to dispose of used menstrual products at home and in schools and about the consequences of throwing them in open or flushing them in toilets. Open discussions on puberty, sex education, menstruation, and so forth should be organized by schools in every class to make students aware. This will solve their unsolved queries by providing them correct knowledge, promote social interaction, and also develop a trust relationship with fellow friends and teachers. School-level health policies should be made by school management committee to promote and educate students regarding health and safety, to ensure adequate water and sanitation facilities, and to protect girl students and staff from bullying and sexual harassment.

*Some Case Studies*

**Case 1 .**

Recently in a school in Tamil Nadu, a 12-year-old school girl of 7th class committed suicide after menstrual shaming. According to her mother, her periods started during a class where she was given a duster cloth to be used as a pad. Then reportedly, she was forced to leave the classroom when her clothes got stained by blood. The next day she did not bear the humiliation and committed suicide due to harassing and torturing by the teacher in the class as written in the suicide note (source: http://www.bbc.com/news/world-asia-india-41107982).

**Case 2 .**

Around 70 girls of the Kasturba School hostel were ordered to remove their clothes by the hostel warden to check for menstrual blood after she found blood stains in the washroom. This shameful act happened in Muzaffarnagar (UP) in March 2017 (source: https://timesofindia.indiatimes.com/city/lucknow/girl-students-told-to-strip-to-check-for-menstrual-blood-up-government-orders-probe/articleshow/57940071.cms).

## 8. Strategies for the Management of Menstrual Waste

**Figure 2:**
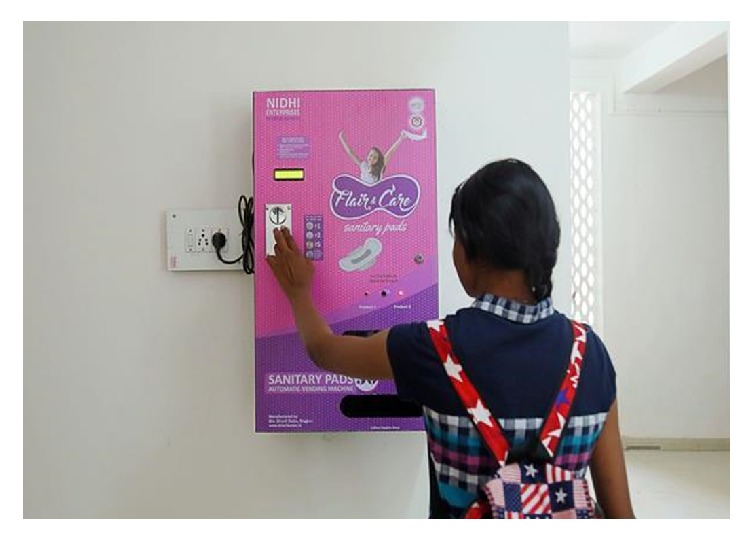
Sanitary napkin vending machine. Source: https://timesofindia.indiatimes.com/city/pune/sanitary-napkin-vending-machines/articleshow/57824878.cms.

1. Disposal of menstrual waste is of major concern as it affects health and environment. There is a need for effective menstrual materials which needs less and cost-effective management.
2. Companies dealing with manufacturing of sanitary pads or other articles should disclose the information on the pads regarding the chemical composition of the pads so that appropriate technologies could be used for their disposal and treatment.
3. Environment-friendly chemicals should be used by manufacturers of sanitary products to stop soil and water pollution and to fasten the decomposition process.
4. Guidance regarding menstrual management to adolescent girls and women is a much needed step. Menstrual hygiene management should be an integral part of education curriculum.
5. Distribution of menstrual products should be free of cost in schools and educational institutes [44]. Recently, instead of subsidizing the menstrual pads, Indian government has imposed 12% GST on them which is not very women friendly (source: http://www.livemint.com/Industry/2Y4RRe0XaJmVduujmsDdXL/GST-rate-on-sanitary-napkins-fixed-at-12.html).
6. The toilets must be designed and built to be girl/women friendly [45]. In Kerala, some schools have installed sanitary napkin vending machines in toilets which are semiautomatic and operate by inserting a coin in it. It contains 30–50 sanitary napkins to meet the emergency needs of the girls/women in schools (Figure 2).
7. There should be a separate collection system for the menstrual wastes without affecting the privacy and dignity of women. Specific sanitary dispensers to collect menstrual waste should be installed.
8. There should be sufficient space for washing, cleaning private parts and hands and for changing or dealing with stained clothes. To fulfil these requirements, there must be water availability, toilet paper, dustbin, and a sink to wash menstrual products.
9. Dustbins should be covered by lid and emptied from time to time to keep the toilets clean from flies, mosquitoes, and bad odour.
10. Covered containers and dustbins have advantage of hiding the waste being seen by others. They are installed in a place that offers privacy [21].
11. Gloves and proper safety tools should be provided to the cleaners so that they are not exposed to pathogenic organisms and harmful gasses.
12. Government should introduce new rules for the safe disposal and treatment of menstrual wastes as they have made for solid or biomedical wastes. Appropriate policy and legal framework is necessary for the management of menstrual wastes.
13. Government and non-government organizations should come forward for making the people aware of management of menstrual wastes. Government should give the funds to the Municipal Corporation or NGOs for the construction of women friendly toilets.
14. Health implications of menstrual wastes should be properly investigated. Financial support should be given to the institutions to carry out the research in the management of menstrual wastes.
15. Scientific research should be encouraged for the most suitable techniques of disposal of sanitary pads or other menstrual products.
16. Allocation of budget in schools to support menstrual hygiene management studies should be conducted.
17. Collaborative efforts (trash bins) should be made.
18. Incinerators are a better option for disposal but should be operated in a controlled environment so that harmful gasses emitted will not harm larger area.

## 9. Better Ways/Ideas of Disposing Menstrual Wastes

### 9.1. Incinerators

**Figure 3:**
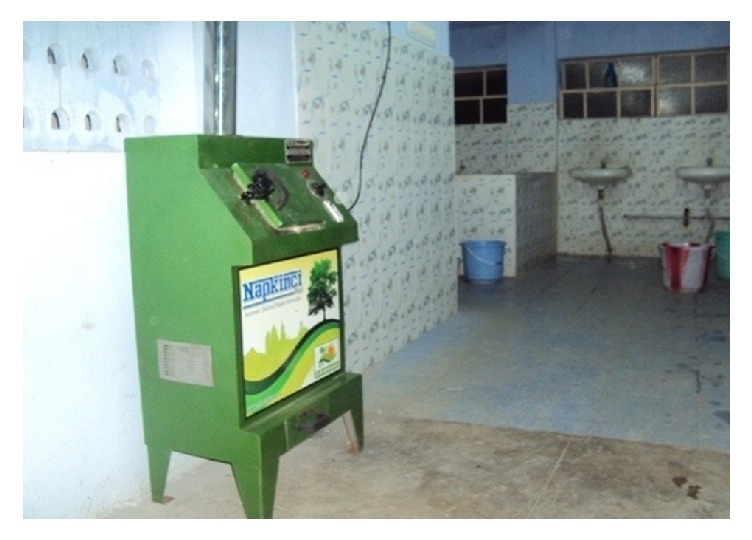
Incinerator installed in the toilet for easy sanitary products disposal. Source: http://www.vendingbiz.in/sanitary-napkin-incinerators-napkinci-maxi-1902444.html.

If incinerators are used according to ecofriendly guidelines they create less pollution. They should be operated at certain specific temperature around 800°C so that they emit less harmful gasses. They should be installed in schools, institutions, and slum areas and at community level (Figure 3).

### 9.2. Latrines with Chutes

These are special kind of toilets in which a shoulder level Chute was made in the usual deep pit. A chemical agent was added to the pit five times in a month to enhance the decomposition process of used napkins.

### 9.3. Reusable Cloth Pads

Using these reusable cloth pads is a better option as they have less chemical and plastic content. So they are easily decomposable as compared to other commercial products.

### 9.4. Biodegradable Products

Commercial sanitary product manufacturing companies must manufacture products having lesser chemical and plastic content. Pads made from bamboo fibre, banana fibre, water hyacinth, and sea sponges should be encouraged.

### 9.5. Clay or Cemented Incinerators

**Figure 4:**
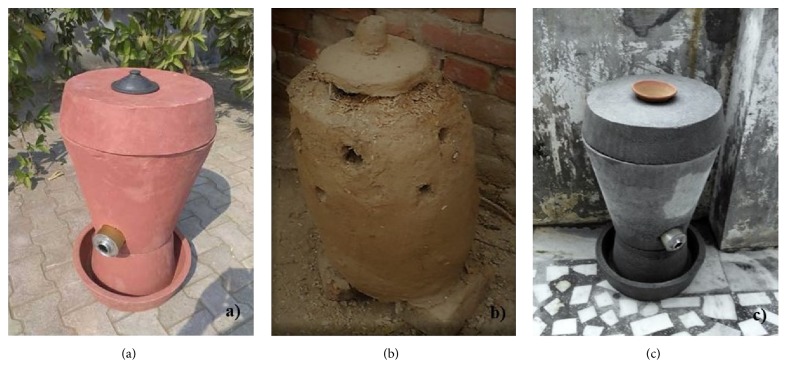
Incinerators used to dispose of menstrual waste in rural areas of India: (a) clay incinerator (http://www.ecoideaz.com/innovative-green-ideas/ashudhinashak-clay-incinerators-for-sanitary-napkins); (b) mud incinerator (https://www.thebetterindia.com/87876/master-art-deal-with-menstrual-waste/); (c) cement incinerator (http://www.ecoideaz.com/innovative-green-ideas/ashudhinashak-clay-incinerators-for-sanitary-napkins).

Clay and cement incinerators used in Gujrat villages by “Vatsalya Foundation” are a welcomed step in menstrual hygiene management. A lady named “Swati” designed this incinerator and named it “Ashudhinashak” which burns many sanitary napkins at a time without creating any smoke. This ecofriendly and cheap innovation is appreciated by rural women who found difficulty in disposing them (Figure 4).

### 9.6. Better Disposal Techniques

Special covered bins should be installed to handle menstrual waste. Disposal bags should be provided by manufacturing companies with color indication for disposing these products. These bags should be freely distributed among schools and institutions. Menstrual waste should not be disposed of along with domestic waste. Pads should be properly wrapped in newspaper and then thrown in the dustbins. By this it should also be safe for rag pickers as it does not expose them to any disease-causing pathogens.

## 10. Conclusions

Menstrual hygiene should be promoted by implementing a course on menstruation and menstrual hygiene management. Teachers should be educated and trained to impart knowledge about menstruation and menstrual hygiene management among students. Social and electronic media also play an important role to make the girls and women aware about the latest menstrual products, different manufacturers, government policies, and so forth. Subsidies should be given on menstrual products so that every girl/women can afford them easily. Non-government organizations should come forward to educate rural people about menstruation, menstrual hygiene management, importance of toilets at homes, hand washing, diseases related to reproductive tract due to poor hygiene, and so forth. Emphases should be given on the use of reusable sanitary or cloth pads to overcome the problem of disposal. Girls and women should be aware of the consequences of disposing used menstrual products in open or flushing them in toilets. Dustbins with proper lids should be placed in the toilets. If possible, incinerators should be installed at homes, schools, and community levels. This study reveals that lack of privacy is a major concern both in household and in schools. Also, ignorance, misconceptions, unsafe practices, and illiteracy of the mother and child regarding menstruation are the root causes of many problems. So, there is a big need to encourage adolescents at school levels to practice safe and hygienic behaviors.
